# Supplementary material for: Cost analysis of treating cardiovascular diseases in a super-specialty hospital
Source: PLoS One. 2022 Jan 5;17(1):e0262190. doi: 10.1371/journal.pone.0262190 (PMC8730466; doi:10.1371/journal.pone.0262190)
Supplement: Attachment — Submitted filename: Response to reviewers.docx [file pone.0262190.s001.docx]

**Response to reviewers – Point wise**

| Reviewer | Observation | Response | Line number |
| --- | --- | --- | --- |
| Academic editor | 1. To respond accurately to the Reviewers’ requests | Yes | Throughout the article |
|  | 2. To improve the methodology of the proposed study | Methodology have been improved as suggested, wherever possible. | In methodology section |
|  | 3. To highlight how the results of the study can be valid in contexts other than the one where the study was carried out | Amended and supported by literature | 260-270 |
|  | 4. To improve the bibliography by adding more recent studies on the subject, particularly researches carried out in Developing Countries other than India. | Recent studies have been added, though there are not many studies similar to the submitted study carried out in developing countries. Findings of a systematic review carried out in LMIC countries in year 2018 has been added. Literature reveals the heterogenicity in the methodology of various cost analysis carried out in developing countries. | 224-230 |
|  | 5. To explain all the acronym used (CTVS, SAARC, INR, PKR) and in particular:  In the abstract the acronym CVDs at line 41.  Line 86 and 95-96 : clarify the observed sample and the criteria chosen to define the sample  Line 102 add the acronym CII after Cost Inflation Index  In table 1 explain the acronym CSSD. | Acronyms have been explained. | Respective lines |
| Additional requirements. | 1. Please ensure that your manuscript meets PLOS ONE's style requirements, including those for file naming | Formatting for manuscript, authors details, images, cover letter etc. has been done as per PLOS ONE’s style. | Throughout the article |
|  | 2. Please provide additional details regarding participant consent. In the ethics statement in the Methods and online submission information, please ensure that you have specified whether consent was written or verbal/oral. If consent was verbal/oral, please specify: 1) whether the ethics committee approved the verbal/oral consent procedure, 2) why written consent could not be obtained, and 3) how verbal/oral consent was recorded. If your study included minors, please state whether you obtained consent from parents or guardians in these cases. If the need for consent was waived by the ethics committee, please include this information | There was some ambiguity and erroneous information, which has been made clear in the manuscript file at relevant places. | 95-100  295-7 |
|  | 3. In your Methods section, please provide a justification for the sample size used in your study, including any relevant power calculations (if applicable) | Justification for sample size has been added. | 94-98 |
|  | 4.Thank you for stating the following financial disclosure:   "unfunded" |  |  |
|  | At this time, please address the following queries:  a) Please clarify the sources of funding (financial or material support) for your study. List the grants or organizations that supported your study, including funding received from your institution.  b) State what role the funders took in the study. If the funders had no role in your study, please state: “The funders had no role in study design, data collection and analysis, decision to publish, or preparation of the manuscript.” c) If any authors received a salary from any of your funders, please state which authors and which funders. d) If you did not receive any funding for this study, please state: “The authors received no specific funding for this work.” | Funding information has been added as  “The authors received no specific funding for this work.” | 301 |
|  | 5. We note that you have referenced (ie. Bewick et al. [5]) which has currently not yet been accepted for publication. Please remove this from your References and amend this to state in the body of your manuscript: (ie “Bewick et al. [Unpublished]”) as detailed online in our guide for authors <http://journals.plos.org/plosone/s/submission-guidelines#loc-reference-style> | All unpublished resources have been removed | Throughout the article |
| Reviewer #1: | The article needs substantial changes. In order to estimate costs of different procedures an activity-based costing approach should be adopted. This means that you should split clearly in the manuscript the three different steps for cost estimation: - identification of resources; - measurement; - valorization  as per the identification step, you should show flow charts depicting the entire process of take-in - charge, from admission to discharge. Moreover, you should identify clearly where and when each activity takes place. I would suggest to focus only on one or two procedures within the cardiovascular domain.  Finally, you should assess the variability and the generalizability of your results to other context. A montecarlo simulation would be very useful in this case. | Included in the limitation of the study, as activity-based costing was not possible due to data availability. It is also pertinent to mention that this research was carried out as part of MD programme requirement for completion and was constrained by availability of resources.  Same has been mentioned in methodology section and in discussion as well.  Patient care flow added | 107-109  221-222  274-275  Figure 1 |
| Reviewer #2: | 1 - No reporting guideline has been explicitly used in the study. Unfortunately, journal policies state that articles should adhere to appropriate reporting guidelines and community standards for data availability. I suggest to identify a guideline of choice (i.e. STROBE) and follow it in the reporting. | Research articles mostly adheres to the various parameters as described in STROBE guidelines, though no reporting guideline has been used. Please suggest if any further changes are to be made. |  |
|  |  |  |  |
|  | - METHODOLOGY |  |  |
|  | 2 - L.86: It is claimed that "Study did not involve any patients" - however, it is stated in several instances that a sample of 100 patients was taken for data modeling. Authors should clarify the meaning of this sentence. Some ambiguity is present in other section of the paper aswell (see L95-96 "no involvement of patients or public, vs. L 234 "The study sample was limited to patients admitted...") | It has been clarified and made unambiguous |  |
|  | 3 - L. 87: It is claimed that "ethical guidelines as deemed appropriate for this study were adhered". Please specify the guidelines the Authors are referring to. | Ethical guidelines as prescribed the | 87-89 |
|  | 4 - L. 93-94: "A total of 100 admitted patients of various CVDs were enrolled in the study using prevalence-based sampling and were followed up till discharge". This approach of taking data from a sample in order to draw conclusions should be accompanied by more rigorous definition of sample size calculation. Authors should clarify the reason that exact number of patients was deemed necessary and sufficient for correct study conduction. | Explained.  It is also pertinent to mention that this research was carried out as part of MD programme requirement for completion and was constrained by availability of resources. | 95-99 |
|  | 5 - L 94-95: Please give reasoning for exclusion criteria | Reasoning for exclusion have been added | 100-101 |
|  | RESULTS |  |  |
|  | 6 - Since data from the sample is taken in order to draw more general conclusions on procedures costs and to compare this against other literature results, it would be more appropriate to clarify the uncertainty level in the study, by indicating confidence intervals for calculated values, and - if the authors deem it feasible - by performing some form of sensitivity analysis. | Sensitivity analysis was not carried out and may require sometime to perform the same, if possible, on given data set. |  |
|  | - DECLARATIONS |  |  |
|  | 7 - It is claimed that "Consent to participate was taken for studying medical records". However, no statement of patient involvement is declared. | Correction has been done to the same as it was not properly clarified. | 95 – 104  295-296 |
